# Diagnosing Growth Hormone Deficiency in Adults

**DOI:** 10.1155/2012/972617

**Published:** 2012-07-26

**Authors:** Nigel Glynn, Amar Agha

**Affiliations:** Department of Endocrinology, Beaumont Hospital, RCSI Medical School, Dublin 9, Ireland

## Abstract

Adult growth hormone (GH) deficiency is a recognised syndrome associated with adverse phenotypic, metabolic, and quality-of-life features which improve in many patients when GH is substituted. The appropriate selection of patients at risk of growth hormone deficiency (GHD) is the crucial first step in arriving at a correct diagnosis. Although multiple diagnostic modalities are available including a 24-hour serum GH profile, stimulated GH levels, and insulin-like growth factor-1 (IGF-1) levels, the use of dynamic tests for GH reserves is required in most cases. This paper discusses the utility and drawbacks of the various testing modalities with reference to international guidelines. Regardless of the test chosen, clinical pitfalls including age and obesity must be taken into account. In addition, there is considerable analytical variation in the biochemical measurements of GH and IGF-1 which must be considered before making a diagnosis of GHD in adulthood.

## 1. Introduction

Severe growth hormone deficiency (GHD) in adults can give rise to several abnormalities. Body composition is altered due to increased fat mass and reduced muscle mass. Exercise capacity is reduced, and quality of life is impaired. The plasma lipid profile is unfavourable, and cardiovascular morbidity may be increased [1]. A growing recognition of this clinical syndrome in the last 20 years has led to the therapeutic use of growth hormone (GH) replacement in adults with severe GHD. This treatment is now available in approximately 80 countries worldwide and has been shown to improve many abnormal parameters [2–5]. GHD is established on both clinical and biochemical criteria, but despite significant advances in our understanding of adult GHD, accurate diagnosis remains challenging. Selecting the appropriate patient, performing a reliable diagnostic test, and understanding the clinical caveats as well as the analytical limitations are the crucial steps.

Consensus guidelines for the diagnosis of adult GHD have been published by professional societies [6–9]. While helpful, recommended diagnostic criteria are not necessarily universally applicable. Problems exist with the performance of some diagnostic tests in terms of accuracy, reproducibility, and resources required. The interpretation of test results may pose further challenges due the variability of current biological assays.

This paper will summarise the current evidence for the appropriate selection of adult patients at risk of GHD, the strengths and limitations of available diagnostic tests, and the characteristics of currently available assays for GH and IGF-1.

## 2. Clinical Context

Adults with GHD comprise two distinct groups–those with a prior diagnosis of GHD in childhood and those who acquire GHD in adulthood due to hypothalamic-pituitary disease. International guidelines consistently advocate that patients with idiopathic childhood-onset GHD should undergo repeat assessment once final adult height is achieved following GH withdrawal for a few months. Many such children will have normal adult GH reserve when retested in adulthood and ongoing GH replacement is not necessary. Children with severe GHD and additional pituitary hormone deficiencies secondary to organic pituitary disease such as craniopharyngioma do not require retesting in adult life [10, 11].

Adult-onset GHD is an uncommon disorder, but the symptoms are subtle and common-place, including fatigue, poor exercise capacity, abdominal obesity, and impaired psychosocial function. Essentially there is no pathognomonic feature. This contrasts with childhood-onset GHD where growth failure acts as a useful biological marker of GHD. In addition, the majority of adults with GHD have deficiencies of other pituitary hormones, further complicating the clinical picture. We cannot therefore rely on symptoms alone for case detection. Identifying patients at risk of GHD such as those with hypothalamic pituitary disease, cranial radiotherapy, head injury, other clinically or biochemically detectable pituitary hormone abnormalities is crucial.  Table 1 outlines the patient groups in whom testing for GHD is recommended. Replacement of GH in deficient adults improves body composition, exercise capacity, cardio-metabolic parameters, bone health and quality of life [1].

## 3. Testing for GH Deficiency

Multiple tests are available for the diagnosis of GHD in adulthood and debate still exists about the most appropriate test. The availability of multiple testing modalities emphasises the complexities involved in making an accurate diagnosis and the need to individualise testing for each patient's clinical circumstances. The “ideal test” will provide clear separation between normal and GHD patients even allowing for factors than may attenuate GH secretion such as age and obesity (see the following).

### 3.1. 24 Hour GH Secretion

In adults, the 24-hour integrated GH profile shows considerable overlap between healthy and GH deficient subjects when using a polyclonal radioimmunoassay to measure GH [12]. Better separation of GHD and normal subjects can be achieved by using a highly sensitive assay for GH [13]. However, this testing method requires frequent sampling over a 24-hour period, which is highly time and resource consuming. Twenty-four-hour urinary GH excretion lacks adequate specificity in separating patients with GHD from normal controls particularly over the age of 40 years. The test yields a sensitivity of 90% but the specificity ranges from 79% for patients under 40 years to 36% for those over 60 years [14].

### 3.2. Serum Insulin-like Growth Factor-1 (IGF-1) Level

IGF-1 is a peptide hormone that mediates most of the biological actions of growth hormone. Circulating IGF-1 is principally composed of endocrine IGF-1 produced in the liver under GH stimulation. A small amount of autocrine IGF-1 is also produced in peripheral tissues such as bone and can be controlled by other factors released from surrounding cells. IGF-1 has a very high affinity for binding proteins (IGFBPs) and circulates in a ternary complex, bound to IGFBP-3 and the acid-labile subunit. It exerts its effect by activation of the IGF-1 receptor which is widely distributed in many tissues [15].

The value of serum IGF-1 and IGF binding protein-3 (IGFBP-3) in the diagnosis of GH deficiency is a matter of contention among endocrinologists. While serum IGF-1 levels less than 2 standard deviation (SD) below the age-matched mean, in a well-nourished adult with pituitary disease, is highly suggestive of GHD [16], it is clear that serum IGF-1 and or IGFBP-3 can be normal in patients with undisputed GHD. Various investigators have reported normal IGF-1 values in 37–70% of GH deficient adults [12, 14, 17, 18]. Further studies, however, showed that age, the time of onset of GHD, and the degree of hypopituitarism, all had a significant influence on serum IGF-1 levels—sometimes expressed as standard deviation scores (IGF-1 SDS) or  *Z*  scores. In the study by Aimaretti et al., 70% of GHD adults under the age of 40 years had a serum IGF-1 level below the age-related 3rd centile, but the corresponding percentage for those over the age of 40 was only 35% [19]. In a large retrospective analysis of patients with GHD from the KIMS database, Lissett et al. found that 86% of patients with childhood-onset GHD compared to 52% with adult-onset GHD had serum IGF-1 SDS less than −2 [20]. The latter study also identified gender, BMI, and number of additional pituitary hormone deficiencies as factors which influence serum IGF-1 SDS. While recognising the above-mentioned caveats, it is now generally accepted that, in well-nourished patients without liver disease, a low IGF-1 in the presence of 3 or more anterior pituitary hormone deficiencies provides very strong evidence of GHD. Further testing in this context is optional [7, 16]. However, for many patients with suspected GHD, a provocative test of growth hormone reserve is required. In addition, since the presence of other pituitary hormone deficiencies is the strongest predictor of GHD and no provocative test has 100% specificity, it is recommended that adults patients who appear to have isolated GHD undergo two provocative tests to confirm the diagnosis, particularly if the serum IGF-1 is not low.

### 3.3. Dynamic Tests of GH Secretion

International consensus guidelines have converged around the insulin tolerance test and the growth-hormone releasing hormone (GHRH) + arginine test (combined test) as the best available test of GHD in adults, providing sufficient sensitivity and specificity to establish a reliable diagnosis when appropriate cutoffs are used. The glucagon stimulation test is a second-line test but is nonetheless well validated for assessing GH secretory capacity when first line tests are unavailable or contra-indicated. Other tests are available but less well validated (see the following).

#### 3.3.1. Insulin Tolerance Test

Hypoglycaemia is a potent stimulus of GH and ACTH-cortisol secretion [21]. This test measures GH reserve by inducing hypoglycaemia with a bolus of intravenous insulin (0.15 units/kg). GH levels are measured every 15–30 minutes for two hours. Following an adequate venous blood glucose nadir of 2.2 mmol/L, a peak GH response of less than 5 ng/mL using a polyclonal radioimmunoassay suggests GHD while a peak of less than 3 ng/mL indicates severe GHD [6, 7, 12]. The latter cutoff provides sufficient separation of normal and hypopituitary subjects even allowing for conditions that result in reduced GH secretion such as age and obesity and is the indication for considering GH replacement in adults [22]. Patients should be adequately replaced with the other hormones before the test is performed.

While the insulin tolerance test is considered the “gold-standard,” it is not a perfect test. It can be safely conducted in experienced centres [23] but is contraindicated in patients with a history of seizures or heart disease. Also, it is unpleasant for the patient who requires hospital admission and close medical supervision, and adequate hypoglycaemia is not always achieved [24]. This consumes considerable healthcare resources and reduces its appeal among some endocrinologists, as illustrated in a recent US study which found that only 11.4% of patients evaluated for GHD underwent an insulin tolerance test [16].

#### 3.3.2. Glucagon Simulation Test

The glucagon stimulation test (GST) is a reliable, safe alternative to the ITT in the diagnosis of GHD [25–29]. Glucagon (1–1.5 mg) is administered intramuscularly and serum samples are taken for GH between 90 and 240 minutes [30]. The GST can also provide co-assessment of ACTH reserve.

The mechanism of glucagon stimulated GH release is not fully understood, although several mechanisms have been proposed [26]. It has been suggested that GH release may result from the drop in plasma glucose later during the test (following its initial rise), but this mechanism is disputed, as the drop in plasma glucose rarely reaches the hypoglycaemic level. Another possible mechanism is by stimulating noradrenaline release, which may stimulate GH secretion via the  *α*-receptor; a suggestion that is, supported by the finding that the administration of  *β*-blockers enhances glucagon-stimulated GH release [31].

Data comparing the GST with the ITT as GH secretagogues have yielded conflicting results. Cain et al. found the GST to be at least as good as the ITT in provoking GH secretion, based on the comparison of overall responses to the two tests [32]. Aimaretti et al. reported, in a large cohort of lean healthy subjects, the third and first centiles normative limits for peak GH response to the GST to be 7.6 ng/mL and 7.1 ng/mL, respectively, compared to 5.3 ng/mL and 3.8 ng/mL, respectively, for the ITT, although the overall response was similar between the two tests [25]. However, the studies by Rahim et al. and Conceição et al. found the ITT to be a more exuberant stimulant of GH than glucagon in healthy subjects [28, 33]; the study by Rahim et al. reported the minimum response to the GST in their healthy subjects to be 11.8 mU/L (comparable to 4 ng/mL). The cut-off limit for the diagnosis of severe GHD using the GST is less well established than that for the ITT, although two studies showed that a cutoff of 3 ng/mL using polyclonal radioimmunoassay to provide reasonable sensitivity and specificity [27, 28]. Berg et al. reported a slightly lower optimal cutoff of 2.5 ng/mL using a modern ultrasensitive chemiluminescent GH assay [29].

The GH response to glucagon may be more likely to be attenuated by age and obesity compared with the ITT [7]. Although the GST is safe, with almost no contraindications, it causes nausea and sometimes vomiting in 15–20% of subjects [25, 26]. In addition it is resource intensive test lasting for three-four hours due to the delayed action of glucagon.

#### 3.3.3. GHRH + Arginine Test

The co-administration of arginine and GHRH (the combined test) is a powerful stimulus for GH production and has gained increasing acceptance as a useful method of diagnosing GHD [34]. This test has been advocated as a suitable alternative to ITT [6, 35–37]. As the amino acid arginine inhibits somatostatin tone, the GHRH-induced GH release is significantly potentiated. An intravenous infusion of arginine (0.5 g/kg body weight) together with an intravenous bolus of GHRH (1 mcg/kg body weight) is administered [30]. Serum samples for GH are then obtained every 15–30 minutes for two hours.

The GHRH + arginine test allows good separation between healthy subjects and those with GH deficiency [37]. However, the cutoff limit for the diagnosis of severe GHD is controversial, with one study suggesting a cutoff of 9 ng/mL [36], while another reporting an optimal cut-off of 4.1 ng/mL [37]. The latter result is supported by a recent study that reported a cut-point of 3.7 ng/mL with an ultrasensitive chemiluminescence-based immunometric assay which conforms to international GH assay guidelines [38]. The difference between these studies may be due to different GH assays used and different characteristics of the control groups—particularly body mass index (BMI). The GH response to the combined test seems to be particularly influenced by BMI, and this is discussed in a later section. This test is safe, and, while half of patients experience flushing, more serious side effects are rare. This test should be avoided in patients with chronic renal failure due the risk of severe hyperkalaemia with arginine infusion [39].

The GHRH + arginine test may give false normal results in patients with GHD secondary to hypothalamic damage, such as those with radiation induced hypopituitarism [40–43]. Hypothalamic injury is apparent earlier than pituitary damage, and therefore direct stimulation of the pituitary by GHRH may give a falsely normal result when compared with ITT. Once 10 years have elapsed following radiotherapy, the two tests appear to perform similarly well.

Other modifications of this test include the combination of GHRH with pyridostigmine or clonidine [44]. In addition, combining GHRH with growth hormone releasing peptides (GHRP-6, GHRP-2, hexarelin) [45] provides a strong stimulus for GH secretion. GHRH + GHRP-6 is now a well-validated test of GH reserve [46] with a cut-off GH concentration of ≥15.0 ng/mL separating normal from hypopituitary subjects. Mahajan and Lightman demonstrated GHRH + GHRP-2 to be a reliable test for GHD despite only measuring a single GH value after 30 minutes [47]. The restricted number of GH measurements makes this an attractive option but the low specificity of 78.6% will temper enthusiasm for this approach. Ghrelin is the natural ligand of the GH secretagogue receptor and may have a role in the diagnosis of GHD in the future. However, more normative data concerning its GH-releasing capacity is required [48].

#### 3.3.4. Arginine Test

Arginine alone (the arginine test) is also used in the assessment of GH reserve. It has been shown to be a less exuberant stimulant for GH secretion than the ITT or the GST [33, 37]. Data on normal GH responses to the arginine test are not very robust; while one study suggested that the third and first centile normative limits to be 2.9 ng/mL and 2.7 ng/mL, respectively [25], another study found a considerable overlap in GH response to arginine between GHD patients and normal controls; 59% of healthy controls had a peak GH response to arginine <3 ng/mL [37]. Reported side effects are rare and include paraesthesia and dry mouth. When compared with other GH stimulation tests including the ITT, the arginine test was ranked the most popular with patients.

Other provocative tests for GH secretion are sometimes used including clonidine alone and the L-dopa tests. They are, however, weak GH secretagogues [33, 37], and their use in adult patients is unreliable.

## 4. Pitfalls in the Diagnosis of GHD

One of the potential caveats in the diagnosis of GHD in adults is the natural decline in GH secretion with age [49]. It has been estimated that GH secretion reduces by approximately 14% per decade from young adult life [50]. However, both 24-hour and arginine-induced GH secretion were found to be lower in elderly patients with pituitary disease than in age-matched healthy controls [51], although there is some overlap between the two groups. A better separation between the two groups may be achieved with the powerful provocative tests, although some physicians are reluctant to use the ITT in the elderly [22]. Colao et al., in a controlled study of over 370 subjects with suspected hypopituitarism, found lower GH cutpoints among elderly patients (>65 years) compared with middle-aged adults after stimulation with GHRH + arginine [49].

Obesity, particularly marked obesity, is associated with blunted GH secretion in response to provocative stimuli [52], and weight loss is associated with the restoration of normal GH production [53]. It has also been suggested that that even mildly increased BMI (25–30 kg/m^2^) can result in diminished stimulated GH production in 13% of healthy subjects [54]. In obesity, serum IGF-1 concentrations are usually normal [55] but some authors reported reduced [56], or even elevated levels in obese children [57]. The pathogenesis of reduced GH secretion in obesity is unknown, but suggested mechanisms include increased hypothalamic somatostatinergic tone or GHRH hypoactivity, hyperinsulinaemia, or elevated circulating free fatty acids [58]. Currently, separate reference data for GH response to most provocative stimuli in obesity are not available. However, Corneli et al. have defined BMI-specific cut-off points for diagnosing adult-onset GHD using GHRH + arginine—11.5 ng/mL for those with BMI < 25 kg/m^2^, 8.0 ng/mL for BMI 25–30 kg/m^2^, 4.2 ng/mL for those with BMI > 30 kg/m^2^ [59].

Additionally, stimulated GH values are affected by oestrogen exposure and phase of the menstrual cycle. GH levels are higher during the luteal phase in comparison with the follicular phase of the cycle [60]. Oral, in contrast to transdermal oestrogen, lowers IGF-1 levels and is associated with increased GH levels [61, 62]. Therefore, one cannot rely on a low IGF-1 to diagnose GHD in women taking oral oestrogen preparations. Adequate pituitary replacement with thyroxine and hydrocortisone are needed for optimal GH production.

## 5. Analytical Considerations

While the measurement of serum GH and IGF-1 concentrations is the cornerstone of the diagnosis of GHD, there are significant analytical problems with the currently available commercial immunoassays. Despite attempts at assay standardisation and the recent increasing use of a highly-sensitive chemiluminescent method for the measurement of GH, there is significant heterogeneity between results obtained in different laboratories [63]. An assay method specific for the 22 kDa isoform of GH is recommended, yet many assays still contain antibodies that detect other circulating forms of GH. Additionally, not all methods have been calibrated with the international reference preparation (IS: 98/574) leading to further interlaboratory discrepancy [64]. Nevertheless the increasing use of monoclonal assays (specific for the 22 kDa isoform of GH) and recalibration with the international standard will overall lead to lower reported GH levels [65]. This has implications for peak GH cut-off levels in provocative testing for GHD and older cut-offs should be adjusted depending on assay performance. GH results are reported in mass units or in international units although the former are now the recommended format [66].

Measurement of IGF-1 also suffers from analytical problems with significant interassay variability. The international reference standard has recently changed (IS: 02/254) but is not universally adopted. Also, accurate measurement of IGF-1 is subject to interference by binding proteins (IGFBPs), and a variety of methods with differing efficacy are used to separate IFG-1 from IGFBPs [67]. More robust normative data with stratification for age groups and gender are required.

Despite the limitations mentioned above, the integrity of GH and IGF-1 measurement can be improved at a local level by defining normal cut-off levels using healthy control subjects from the hospital's catchment population. This will avoid recourse to a reference laboratory in most circumstances.

## 6. Conclusion

Adult-onset GHD is now a well-recognised clinical syndrome and multiple benefits can be accrued from GH replacement. Investigating patients within the appropriate clinical context is important to identify those who may be eligible for treatment. While it is widely accepted that a low IGF-1 value in the presence of multiple pituitary hormone deficiencies provides strong evidence of GHD in adults, most patients will require provocative testing to confirm the diagnosis. Numerous GH secretagogues are available with the insulin tolerance test being the gold standard and the glucagon stimulation test or the GHRH + arginine as acceptable alternatives. GH response to stimulation is both stimulus and assay dependent and can be influenced by factors such as age and BMI. All these variables should be considered when defining severe GH deficiency as an indication for GH replacement.

## Figures and Tables

**Table 1:** Adult patients in whom testing for GHD can be considered.

(i) Those with structural lesions of hypothalamic-pituitary region for example, pituitary adenoma, craniopharyngioma	
(ii) Following surgery to the hypothalamic-pituitary region	
(iii) Those with biochemical evidence of hypopituitarism for example, central hypothyroidism	
(iv) Those with genetic conditions associated with hypopituitarism for example, PIT-1, PROP-1 mutations	
(v) Following cranial irradiation	
(vi) Following moderate to severe traumatic brain injury and possibly aneurysmal subarachnoid haemorrhage	

## References

[1] Thomas JDJ, Monson JP (2009). Adult GH deficiency throughout lifetime. *European Journal of Endocrinology*.

[2] Al-Shoumer KAS, Gray R, Anyaoku V (1998). Effects of four years’ treatment with biosynthetic human growth hormone (GH) on glucose homeostasis, insulin secretion and lipid metabolism in GH- deficient adults. *Clinical Endocrinology*.

[3] Baum HBA, Biller BMK, Finkelstein JS (1996). Effects of physiologic growth hormone therapy on bone density and body composition in patients with adult-onset growth hormone deficiency: a randomized, placebo-controlled trial. *Annals of Internal Medicine*.

[4] Götherström G, Svensson J, Koranyi J (2001). A prospective study of 5 years of GH replacement therapy in GH-deficient adults: sustained effects on body composition, bone mass, and metabolic indices. *Journal of Clinical Endocrinology and Metabolism*.

[5] Widdowson WM, Gibney J (2008). The effect of growth hormone replacement on exercise capacity in patients with GH deficiency: a metaanalysis. *Journal of Clinical Endocrinology and Metabolism*.

[6] Ho KKY (2007). Consensus guidelines for the diagnosis and treatment of adults with GH deficiency II: a statement of the GH Research Society in association with the European Society for Pediatric Endocrinology, Lawson Wilkins Society, European Society of Endocrinology, Japan Endocrine Society, and Endocrine Society of Australia. *European Journal of Endocrinology*.

[7] Molitch ME, Clemmons DR, Malozowski S, Merriam GR, Vance ML (2011). Evaluation and treatment of adult growth hormone deficiency: an endocrine society clinical practice guideline. *Journal of Clinical Endocrinology and Metabolism*.

[8] Gharib H, Cook DM, Saenger PH (2003). American Association of Clinical Endocrinologists medical guidelines for clinical practice for growth hormone use in adults and children—2003 update. *Endocrine Practice*.

[9] Giustina A, Barkan A, Chanson P (2008). Guidelines for the treatment of growth hormone excess and growth hormone deficiency in adults. *Journal of Endocrinological Investigation*.

[10] Nicolson A, Toogood AA, Rahim A, Shalet SM (1996). The prevalence of severe growth hormone deficiency in adults who received growth hormone replacement in childhood. *Clinical Endocrinology*.

[11] Gleeson HK, Shalet SM (2004). The impact of cancer therapy on the endocrine system in survivors of childhood brain tumours. *Endocrine-Related Cancer*.

[12] Hoffman DM, Nguyen TV, O’Sullivan AJ (1994). Diagnosis of growth hormone deficiency in adults. *The Lancet*.

[13] Reutens AT, Hoffman DM, Leung KC, Ho KKY (1995). Evaluation and application of a highly sensitive assay for serum growth hormone (GH) in the study of adult GH deficiency. *Journal of Clinical Endocrinology and Metabolism*.

[14] Bates AS, Evans AJ, Jones P, Clayton RN (1995). Assessment of GH status in adults with GH deficiency using serum growth hormone, serum insulin-like growth factor-I and urinary growth hormone excretion. *Clinical Endocrinology*.

[15] Clemmons DR (2007). Value of insulin-like growth factor system markers in the assessment of growth hormone status. *Endocrinology and Metabolism Clinics of North America*.

[16] Hartman ML, Crowe BJ, Biller BMK, Ho KKY, Clemmons DR, Chipman JJ (2002). Which patients do not require a GH stimulation test for the diagnosis of adult GH deficiency?. *Journal of Clinical Endocrinology and Metabolism*.

[17] Lee SC, Zasler ND, Kreutzer JS (1994). Male pituitary-gonadal dysfunction following severe traumatic brain injury. *Brain Injury*.

[18] Svensson J, Johannsson G, Bengtsson BÅ (1997). Insulin-like growth factor-I in growth hormone-deficient adults: relationship to population-based normal values, body composition and insulin tolerance test. *Clinical Endocrinology*.

[19] Aimaretti G, Corneli G, Baldelli R (2003). Diagnostic reliability of a single IGF-I measurement in 237 adults with total anterior hypopituitarism and severe GH deficiency. *Clinical Endocrinology*.

[20] Lissett CA, Jönsson P, Monson JP, Shalet SM (2003). Determinants of IGF-I status in a large cohort of growth hormone-deficient (GHD) subjects: the role of timing of onset of GHD. *Clinical Endocrinology*.

[21] Fish HR, Chernow B, O’Brian JT (1986). Endocrine and neurophysiologic responses of the pituitary to insulin-induced hypoglycemia: a review. *Metabolism*.

[22] Shalet SM, Toogood A, Rahim A, Brennan BMD (1998). The diagnosis of growth hormone deficiency in children and adults. *Endocrine Reviews*.

[23] Finucane FM, Liew A, Thornton E, Rogers B, Tormey W, Agha A (2008). Clinical insights into the safety and utility of the insulin tolerance test (ITT) in the assessment of the hypothalamo-pituitary-adrenal axis. *Clinical Endocrinology*.

[24] Jones SL, Trainer PJ, Perry L, Wass JAH, Besser GM, Grossman A (1994). An audit of the insulin tolerance test in adult subjects in an acute investigation unit over one year. *Clinical Endocrinology*.

[25] Aimaretti G, Baffoni C, DiVito L (2000). Comparisons among old and new provocative tests of GH secretion in 178 normal adults. *European Journal of Endocrinology*.

[26] Leong KS, Walker AB, Martin I, Wile D, Wilding J, MacFarlane IA (2001). An audit of 500 subcutaneous glucagon stimulation tests to assess growth hormone and ACTH secretion in patients with hypothalamic-pituitary disease. *Clinical Endocrinology*.

[27] Gómez JM, Espadero RM, Escobar-Jiménez F (2002). Growth hormone release after glucagon as a reliable test of growth hormone assessment in adults. *Clinical Endocrinology*.

[28] Conceição FL, da Costa e Silva A, Leal Costa AJ, Vaisman M (2003). Glucagon stimulation test for the diagnosis of GH deficiency in adults. *Journal of Endocrinological Investigation*.

[29] Berg C, Meinel T, Lahner H, Yuece A, Mann K, Petersenn S (2010). Diagnostic utility of the glucagon stimulation test in comparison to the insulin tolerance test in patients following pituitary surgery. *European Journal of Endocrinology*.

[30] Trainer PJ, Besser GM (1995). *The Bart's Endocrine Protcols*.

[31] Mitchell ML, Suvunrungsi P, Sawin CT (1971). Effect of propranolol on the response of serum growth hormone to glucagon. *Journal of Clinical Endocrinology and Metabolism*.

[32] Cain JP, Williams GH, Dluhy RG (1972). Glucagon-initiated human growth hormone release: a comparative study. *Canadian Medical Association Journal*.

[33] Rahim A, Toogood AA, Shalet SM (1996). The assessment of growth hormone status in normal young adult males using a variety of provocative agents. *Clinical Endocrinology*.

[34] Webb SM, Strasburger CJ, Mo D (2009). Changing patterns of the adult growth hormone deficiency diagnosis documented in a decade-long global surveillance database. *Journal of Clinical Endocrinology and Metabolism*.

[35] Ghigo E, Aimaretti G, Gianotti L, Bellone J, Arvat E, Camanni F (1996). New approach to the diagnosis of growth hormone deficiency in adults. *European Journal of Endocrinology*.

[36] Aimaretti G, Corneli G, Razzore P (1998). Comparison between insulin-induced hypoglycemia and growth hormone (GH)- releasing hormone + arginine as provocative tests for the diagnosis of GH deficiency in adults. *Journal of Clinical Endocrinology and Metabolism*.

[37] Biller BMK, Samuels MH, Zagar A (2002). Sensitivity and specificity of six tests for the diagnosis of adult GH deficiency. *Journal of Clinical Endocrinology and Metabolism*.

[38] Chanson P, Cailleux-Bounacer A, Kuhn JM (2010). Comparative validation of the growth hormone-releasing hormone and arginine test for the diagnosis of adult growth hormone deficiency using a growth hormone assay conforming to recent international recommendations. *Journal of Clinical Endocrinology and Metabolism*.

[39] Bushinsky DA, Gennari FJ (1978). Life-threatening hyperkalemia induced by arginine. *Annals of Internal Medicine*.

[40] Darzy KH, Aimaretti G, Wieringa G, Rao Gattamaneni H, Ghigo E, Shalet SM (2003). The usefulness of the combined growth hormone (GH)-releasing hormone and arginine stimulation test in the diagnosis of radiation-induced GH deficiency is dependent on the post-irradiation time interval. *Journal of Clinical Endocrinology and Metabolism*.

[41] Ham JN, Ginsberg JP, Hendell CD, Moshang T (2005). Growth hormone releasing hormone plus arginine stimulation testing in young adults treated in childhood with cranio-spinal radiation therapy. *Clinical Endocrinology*.

[42] Björk J, Link K, Erfurth EM (2005). The utility of the growth hormone (GH) releasing hormone-arginine test for diagnosing gh deficiency in adults with childhood acute lymphoblastic leukemia treated with cranial irradiation. *Journal of Clinical Endocrinology and Metabolism*.

[43] Popovic V, Pekic S, Golubicic I, Doknic M, Dieguez C, Casanueva FF (2002). The impact of cranial irradiation on GH responsiveness to GHRH plus GH-releasing peptide-6. *Journal of Clinical Endocrinology and Metabolism*.

[44] Hoeck HC, Vestergaard P, Jakobsen PE, Falhof J, Laurberg P (2000). Diagnosis of growth hormone (GH) deficiency in adults with hypothalamic-pituitary disorders: comparison of test results using pyridostigmine plus GH-Releasing hormone (GHRH), clonidine plus GHRH, and insulin-induced hypoglycemia as GH secretagogues. *Journal of Clinical Endocrinology and Metabolism*.

[45] Abs R (2003). Update on the diagnosis of GH deficiency in adults. *European Journal of Endocrinology*.

[46] Popovic V, Leal A, Micic D (2000). GH-releasing hormone and GH-releasing peptide-6 for diagnostic testing in GH-deficient adults. *The Lancet*.

[47] Mahajan T, Lightman SL (2000). A simple test for growth hormone deficiency in adults. *Journal of Clinical Endocrinology and Metabolism*.

[48] Aimaretti G, Baffoni C, Broglio F (2002). Endocrine responses to ghrelin in adult patients with isolated childhood-onset growth hormone deficiency. *Clinical Endocrinology*.

[49] Colao A, Di Somma C, Savastano S (2009). A reappraisal of diagnosing GH deficiency in adults: role of gender, age, waist circumference, and body mass index. *Journal of Clinical Endocrinology and Metabolism*.

[50] Toogood AA, O’Neill PA, Shalet SM (1996). Beyond the somatopause: growth hormone deficiency in adults over the age of 60 years. *Journal of Clinical Endocrinology and Metabolism*.

[51] Toogood AA, Nass RM, Pezzoli SS, O’Neill PA, Thorner MO, Shalet SM (1997). Preservation of growth hormone pulsatility despite pituitary pathology, surgery, and irradiation. *Journal of Clinical Endocrinology and Metabolism*.

[52] Williams T, Berelowitz M, Joffe SN (1984). Impaired growth hormone responses to growth hormone-releasing factor in obesity. A pituitary defect reversed with weight reduction. *The New England Journal of Medicine*.

[53] Rasmussen MH, Hvidberg A, Juul A (1995). Massive weight loss restores 24-hour growth hormone release profiles and serum insulin-like growth factor-I levels in obese subjects. *Journal of Clinical Endocrinology and Metabolism*.

[54] Bonert VS, Elashoff JD, Barnett P, Melmed S (2004). Body mass index determines evoked growth hormone (GH) responsiveness in normal healthy male subjects: diagnostic caveat for adult GH deficiency. *Journal of Clinical Endocrinology and Metabolism*.

[55] Maccario M, Gauna C, Procopio M (1999). Assessment of GH/IGF-I axis in obesity by evaluation of IGF-I levels and the GH response to GHRH+arginine test. *Journal of Endocrinological Investigation*.

[56] Iranmanesh A, Lizarralde G, Veldhuis JD (1991). Age and relative adiposity are specific negative determinants of the frequency and amplitude of growth hormone (GH) secretory bursts and the half-life of endogenous GH in healthy men. *Journal of Clinical Endocrinology and Metabolism*.

[57] Loche S, Pintor C, Cappa M (1989). Pyridostigmine counteracts the blunted growth hormone response to growth hormone-releasing hormone of obese children. *Acta Endocrinologica*.

[58] Pontiroli AE, Lanzi R, Monti LD, Sandoli E, Pozza G (1991). Growth hormone (GH) autofeedback on GH response to GH-releasing hormone. Role of free fatty acids and somatostatin. *Journal of Clinical Endocrinology and Metabolism*.

[59] Corneli G, Di Somma C, Baldelli R (2005). The cut-off limits of the GH response to GH-releasing hormone-arginine test related to body mass index. *European Journal of Endocrinology*.

[60] Faria ACS, Bekenstein LW, Booth RA (1992). Pulsatile growth hormone release in normal women during the menstrual cycle. *Clinical Endocrinology*.

[61] Friend KE, Hartman ML, Pezzoli SS, Clasey JL, Thorner MO (1996). Both oral and transdermal estrogen increase growth hormone release in postmenopausal women - A clinical research center study. *Journal of Clinical Endocrinology and Metabolism*.

[62] Weissberger AJ, Ho KKY, Lazarus L (1991). Contrasting effects of oral and transdermal routes of estrogen replacement therapy on 24-hour growth hormone (GH) secretion, insulin-like growth factor I, and GH-binding protein in postmenopausal women. *Journal of Clinical Endocrinology and Metabolism*.

[63] Pokrajac A, Wark G, Ellis AR, Wear J, Wieringa GE, Trainer PJ (2007). Variation in GH and IGF-I assays limits the applicability of international consensus criteria to local practice. *Clinical Endocrinology*.

[64] Clemmons DR (2011). Consensus statement on the standardization and evaluation of growth hormone and insulin-like growth factor assays. *Clinical Chemistry*.

[65] Bidlingmaier M, Strasburger CJ (2007). Growth hormone assays: current methodologies and their limitations. *Pituitary*.

[66] Trainer PJ, Barth J, Sturgeon C, Wieringa G (2006). Consensus statement on the standardisation of GH assays. *European Journal of Endocrinology*.

[67] Frystyk J, Freda P, Clemmons DR (2010). The current status of IGF-I assays—a 2009 update. *Growth Hormone and IGF Research*.

